# The ASIA syndrome: basic concepts

**DOI:** 10.31138/mjr.28.2.64

**Published:** 2017-06-27

**Authors:** Abdulla Watad, Kassem Sharif, Yehuda Shoenfeld

**Affiliations:** 1 Department of Medicine ‘B’,; 2 Zabludowicz Center for Autoimmune Diseases, Sheba Medical Center, Tel-Hashomer, Israel; Sackler Faculty of Medicine, Tel-Aviv University, Tel-Aviv, Israel

**Keywords:** ASIA syndrome, autoimmune/inflammatory syndrome induced by adjuvants, adjuvants, vaccine, autoantibodies, silicone, autoimmunity

## Abstract

The autoimmune/inflammatory syndrome induced by adjuvants (ASIA), also known as Shoenfeld’s syndrome, encompasses several autoimmune conditions/phenomena that are induced following the exposure to substances with adjuvant activity. The disease spectrum is heterogeneous in respect to clinical presentation as well as severity of the clinical manifestations. Adjuvants are included in vaccination formulations for their immunogenic properties. Despite being generally well tolerated, safe and effective, some genetically predisposed individuals can develop generalized non-specific constitutional symptoms, autoantibody production, new onset, or worsening of disease presentation. In this review, we focus on the current knowledge presented in the literature on ASIA syndrome, increasing physician awareness about the basic concepts of ASIA syndrome and highlight the devastating amount of data accumulated in the last few years concerning the relationship between various adjuvants and autoimmunity.

## INTRODUCTION

Autoimmune/Inflammatory syndrome induced by adjuvants (ASIA) is a disease entity that was first introduced by Shoenfeld et al. in 2011. ^1^ The authors have proposed several major and minor criteria that may aid in the diagnosis of ASIA syndrome ( **Table 1** ). It constitutes a set of closely related immune mediated diseases that share a common clinical picture as well as a history of a previous exposure to an adjuvant agent. ^2^ These common denominators were prominent in individuals who developed macrophagic myofasciitis syndrome (MMF), post vaccination phenomenon, Gulf War syndrome (GWS), and siliconosis. ^1^ Post-vaccination phenomena and MMF are thought to mainly develop following the use of aluminum-based adjuvants. ^2^ In contrast, the incidence of GWS was contributed to squalene adjuvant exposure. ^4^ From a clinical standpoint, these four diseases present with classical constitutional manifestations that include myalgia, arthralgia, chronic fatigue and dry mouth as well as neurological manifestations such as cognitive disturbances, memory loss and neurologic disabilities. ^5^ Resembling other autoimmune disease entities, the etiopathogenesis of these conditions involves a multifactorial interplay between environmental factors and genetic predisposition as noted by the association with certain HLA haplotypes. ^6^ Since its emergence as a disease entity, more than 4000 documented cases of ASIA syndrome have been reported with various clinical severity and diverse history of adjuvant exposure. ^7^ In this review we sought to summarize the current literature on ASIA syndrome and its relation to vaccines and silicone. We aim to highlight basic concepts in order to increase physician awareness and therefore facilitate early diagnosis and prevent the exposure to adjuvants to those subjects with high risk for autoimmunity.

**Table 1.** Suggested criteria for the diagnosis of ‘ASIA’

**Major Criteria:** **Exposure to an external stimuli (Infection, vaccine, silicone, adjuvant) prior to clinical manifestations.** **The appearance of ’typical’ clinical manifestations:** - Myalgia, Myositis or muscle weakness - Arthralgia and/or arthritis - Chronic fatigue, un-refreshing sleep or sleep disturbances - Neurological manifestations (especially associated with demyelination) - Cognitive impairment, memory loss - Pyrexia, dry mouth **Removal of inciting agent induces improvement** **Typical biopsy of involved organs**
**Minor Criteria:** **The appearance of autoantibodies or antibodies directed at the suspected adjuvant** **Other clinical manifestations (i.e. irritable bowel syn.)** **Specific HLA (i.e. HLA DRB1, HLA DQB1)** **Evolvement of an autoimmune disease (i.e. MS, SSc)**

## THE ROLE OF ADJUVANTS

Adjuvants are immunological molecules that function through potentiating antigen specific immune responses. ^8^ While adjuvants themselves do not mount an immune response, they aid in the production of a robust reaction against their inoculated antigens. Adjuvants are frequently employed in the field of medicine, more specifically in vaccination production. ^8^ The utilization of adjuvants contribute towards a heightened immunogenicity response resulting in a reduced frequency and amount of vaccination required to attain adequate preventive immunity. ^9^ Additionally, adjuvants act as a depot facilitating a prolonged antigen presence in the blood, as well as a delivering vehicle carrying inoculated antigens to lymphocyte rich areas including lymph nodes. ^10^ Both of these mechanisms strengthen the resultant immune response; a response that is not strongly provoked otherwise. ^10^

Broadly, adjuvants influence both the adaptive and the innate arms of the immune system via various mechanisms which results in initiation as well as substantiation of immune response by the activation of pattern recognition receptors, more specifically toll like receptors (TLR), NOD-like receptors (NOR), and C-type lectin receptors. ^11^ The activation of these molecules subsequently leads to a recruitment and downstream protein activation that contributes toward cytokine generation. ^11^ Furthermore, adjuvants promote certain aspects of dendritic cells chemotaxis and antigen presenting cells activation which further potentiates antigen transfer to B-cell and T-cell rich environments and thus enhancing adaptive immune response to antigen. ^12^

According to Edelman, adjuvants can be classified to three distinct groups; active immune stimulants that potentiate immune response to the antigen, carriers which enable T-cell help, and vehicle adjuvants which inoculate the antigens and facilitated immune response activation. ^13^

Adjuvants can usually be produced from a myriad of substances including for example: oils, mineral salts, lipopolysaccharides and peptidoglycan. ^11^

In spite of its beneficial effects, the application of adjuvants in medicine was shown to induce non-specific generalized constitutional musculoskeletal symptoms. ^12^ Moreover, in genetically susceptible individuals, adjuvant administration can lead to overt autoimmune disease induction, as well as autoantibody production. ^14,15^ These incidences were repeatedly reported and documented by observational studies and experimental animal studies following adjuvant administration. ^16,17^

## POST-VACCINATION EFFECT AND ASIA

The breakthrough in vaccination development is one of the greatest public health movements in the last century. Vaccinations provide protection against the formerly known infectious diseases that in many cases led to disabling consequences as well as high morbidity and mortality rates. ^18^ Although generally well tolerated, occasionally vaccine administration led to autoimmune manifestation induction as well as autoantibody production. ^19^ Further, patients with preexisting rheumatologic diseases witnessed a worsening in their disease process subsequent to vaccinations. ^20^

The post vaccination phenomena have been postulated to be largely attributed to the immunological characteristics of adjuvants that are concurrently administered with vaccination. ^20^ Adjuvant formulations have been widely studied with the intention of finding preparations with high stability, strong immunogenicity, and sufficient bioavailability while still being well tolerated. ^21^ Aluminum salts, mostly aluminum phosphate or hydroxide, are one of the most frequently used adjuvants which were shown to enhance antigen presentation, complement activation, innate immune system stimulation and T-helper cells (TH)1 and TH2 activation. ^22^ Vaccines with aluminum based adjuvants where associated with post vaccination phenomena and ASIA-related symptoms. ^23^ Animal models showed a higher seroconversion and antibody levels for systemic lupus erythematosus (SLE) following hepatitis B virus (HBV) vaccination in genetically susceptible mice. ^24^ Moreover, alum-based adjuvant administration in mice led to the development of Sjögren-like disease resulting in the development of higher antibody levels, decreased saliva volume, and documented histological inflammatory acinar response. ^25^

Comparable to the results witnessed in the animal models, multiple studies have been carried with the purpose of documenting autoimmune adverse effect patterns associated with vaccination administration. ^26,27^ These studies were based on safety surveillance data made available by database system in the United States, which is known as vaccine adverse event reporting system (VAERS). A case control study revealed that over a 6 year vaccination period with the quadrivalent HPV vaccine, a reported increased risk of arthritis, vasculitis, SLE and neurological conditions were documented as compared to the general population. ^26^ These effects mostly occurred on average 1 to 7 weeks after vaccination. The quadrivalent HPV vaccine is prepared by inoculation of virus-like particles of the constituent four serotypes HPV type 6,11,16 and 18 in aluminum-based adjuvant. ^26^ Numerous other associations between vaccination and autoimmune phenomenon have been documented of which include: Guillain-Barre syndrome outbreak after H1N1 vaccination and HBV vaccination, idiopathic thrombocytopenic purpura (ITP) in subsequence to Measles-mumps-rubella (MMR) and varicella zoster vaccination, arthritis following diphtheria tetanus-pertussis (DTP) and MMR vaccinations, ^27^ and influenza vaccine preceding Hashimoto’s thyroiditis. ^28^ Furthermore, multiple cases of giant cell arthritis (GCA) were documented in otherwise healthy individuals who received adjuvanted influenza vaccination during the previous three months. ^29^

Other than the postulated role of adjuvants in the resultant autoimmune response, other vaccine components exampled by neomycin and polymyxin have been implicated in mounting certain immunological responses, which occur as a result of influencing certain immunologic responses. ^30^

Several mechanisms have been suggested by which infections can induce autoimmune diseases. One of these mechanisms is the molecular mimicry that requires both antigen and immunologic adjuvant. This phenomenon normally induces autoimmune diseases in those with genetically predisposition for autoimmunity. The demyelinating disease induced by the HBV vaccine is a good example to illustrate and clarify these phenomena. ^31^ When a structural similarity exists between some viral antigen (or other component of the vaccine) and a self-antigen. This similarity may be the trigger to the autoimmune reaction. Other mechanism by which vaccines may induce autoimmunity is the increase in immune complexes which can in turn cause the vasculitis noted in several cases, or the exacerbation of existing autoimmune symptoms. ^17^ At large, the individual role of each independent complement is still to be elucidated; yet, emerging data supports the notion of the existence of autoimmune response following vaccination administration. Although most epidemiological studies do not indicate apparent vaccine safety concerns, awareness of ASIA syndrome development as well as identification of certain high-risk attributes could prevent such adverse reactions.

## MACROPHAGIC MYOFASCIITIS SYNDROME (MMF) AND ADJUVANT ADMINISTRATION

Another closely related disease in the ASIA syndrome spectrum is MMF, which is noted to develop after certain vaccine administration. As suggested by the disease name, the accumulation of agglomerate forming alum nano-crystalline molecules in the macrophages between muscle fibers leads to autoimmune inflammation and induces non-specific clinical symptoms including myalgia, fatigue, and CNS involvement with reported decreased memory function, disturbed mood, and attention deficit. ^32^ In addition to those constitutional symptoms, close to one-fifth of MMF patients developed a contemporaneous well-defined autoimmune disease, most frequently Hashimoto’s thyroiditis, dermatomyositis, myasthenia gravis, autoimmune myopathy, inclusion body myositis and multiple sclerosis-like demyelinating disease. ^32^ Histologic findings showed a strongly periodic acid-schiff staining macrophages, a prominent CD8 positive infiltrating T cells, and positive hematoxylin staining aluminum crystals. ^33^ In contrast, surrounding myofibers are typically left intact, unless an additional autoimmune disease process is present. In 457 studied MMF patients, Gheradi et al ^3^ documented a mean of 5.3 alum containing vaccine administration, with 85% of the studied sample reporting previous vaccination against HBV. The average latency of symptoms appeared to be around 7 months after vaccine administration. Interestingly, these patients had no previous distinctive aluminum exposure other than their exposure to adjuvant-associated alum compounds including Hepatitis A virus, HBV and tetanus toxoid vaccines. ^3^ Such findings stand in agreement with the increased vaccination against HBV during the same period as well as the use of intramuscular route of HBV vaccination, replacing the older subcutaneous route of administration. ^4^

The integral role of genetic susceptibility and MMF development was supported by the relative rarity of the condition as compared to the widespread usage of aluminum hydroxide. In fact, a case control study involving 9 MMF patients and 230 controls demonstrated an increased HLA-DRB1*01 haplotype presence among patients with MMF as compared to healthy control (Odds ratio: 9.8, 95%CI:2.0–62.0, p<0.03). ^34^

## GULF WAR SYNDROME (GWS) AND ASIA

The appearance of systematic constitutional symptoms including fatigue, muscle weakness and arthralgia in military veterans and civil workers during the Gulf War period led to the emergence of GWS entity. To explain this phenomenon, certain exposure to hazards were identified during this war including the exposure to pyridostigmine, radioactive munition and military vaccination protocol. ^35^ Remarkably, veterans deployed to war had to be vaccinated with a six shot regimen for anthrax vaccine; a preparation that was adjuvanted with alum and squalene. ^12^

Squalene, which is an oil-based adjuvant, increases antigen uptake by APC and leads to adaptive immune system activation. ^36^ Although safe and well tolerated in humans, exposure to squalene have been shown to be possibly implicated with provoking chronic fatigue syndrome; a response that is postulated to be mediated by TH2-cell response. ^36^ In animal models, squalene exposure in mice led to the development of immune mediated arthritis in all of the tested subjects, which was detected by the appearance of signs of synovitis including pannus formation, osteolysis and chondrolysis noted in histological examination. ^37^

The relationship between exposure to squalene and GWS development was supported by a case control study involving 144 Gulf War veterans, of which 95% of deployed GWS patients had antibodies to squalene. In the subcategory of GWS patients who received the vaccinations yet did not deploy to service, the presence of squalene antibodies was documented in 100% of these patients. On the other hand, healthy adults, or patients with other autoimmune syndromes, and vaccinated veterans without clinical signs of GWS did not form squalene antibodies. ^38^

While an exact causal relationship still requires elucidation, the evidence of an association between adjuvant administration and GWS is present, a finding that necessitates caution regarding the induced side effects after vaccination administration.

## SILICONE AND ASIA SYNDROME

Silicone as an adjuvant material has been long thought to be an inert material, and therefore has been used for more than 5 decades in medical implants including heart valves, testicular prostheses, intraocular lenses and breast implants. ^39^ Although considered otherwise safe, reports are emerging regarding the development of autoimmune-like phenomena in patients as well as animal models exposed to silicone. ^40^ Silicone as an adjuvant potentiates the immune response by promoting adaptive immune cell proliferation and cytokine release that leads to T cell proliferation and polarization. ^41^ Additionally, silicone is notable for enhancing immnoreactivity by its reaction with components of the connective tissue including mucopolysaccardies and naturally occurring silicone substances. ^42^

As with previously mentioned diseases, exposure to silicone adjuvants led to immunologically-mediated immune responses that manifested as arthralgia, arthritis, malaise and pyrexia, headaches, fatigue and generalized weakness. ^40^

Although the constellation of these symptoms satisfied certain criteria of fibromyalgia and chronic fatigue syndrome, they lacked the presence of the prominent symptoms characterizing those diseases. Moreover, all these symptoms did not fulfill a distinctive rheumatological disease diagnostic criteria.

Several research groups set to investigate the relationship of silicone and the appearance of the autoimmune-associated clinical manifestation. Vasey et al. ^43^ documented a statistically significant increase of the reported constitutional symptoms including pain and fatigue in patients with silicone implants and silicone implant ruptures. In line with this study, a large study conducted by Fryzek et al. ^44^ showed statistically significant reports of constitutional symptoms and rheumatological symptoms in 1546 patients as compared with 2496 controls that underwent breast reduction surgeries. In another study, 69% of women had improvement of constitutional symptoms following silicone implant extraction. ^45^ All of these findings stand in agreement of the possible relationship between silicone and the grouping of these symptoms – ASIA syndrome. Other factors have also been seen to play a role in the development of autoantibodies. In a cohort study, vitamin D levels in silicone-implanted breast patients were shown to be inversely related to antibody levels (Relative risk 3.14, 95%CI: 1.24–7.59). ^46^ This result suggests the possible beneficial role of the immunomodulatory action of vitamin D in preventing the subsequent antibody formation in silicone-implanted patients.

As noted with other autoimmune diseases, a certain genetic predisposition seems to play in disease development. ASIA patients with silicone implants were more likely to possess certain genetic haplotypes including HLA-DR5 and HLA DQ2 when compared to women with breast implants who were symptom-free. ^47^ This awareness to the increased probability and predisposition of this subcategory to silicone implants should warrant their extraction when patients present with this clinical manifestation.

## CONCLUSION

ASIA syndrome, also known as Shoenfeld’s syndrome, has been frequently reported since its emergence as a disease entity. Genetic predisposition and environmental exposure to adjuvants seem to be the largest factors in disease pathogenesis. Due to the presence of reported severe ASIA cases, more efforts should be put towards clarifying and understating the governing relationship between adjuvant administration and autoimmunity. The awareness of the diseases as well as its manifestations is essential for the prevention and the treatment of cases that develop subsequent to adjuvant exposure.
